# Consistency of Mini-Nutritional Assessment short form and Nutritional Risk Screening 2002 in nutritional evaluation of diabetic foot patients

**DOI:** 10.3389/fnut.2025.1596193

**Published:** 2025-06-04

**Authors:** Qian Ran, Xili Zhao, Weiwei Xu, Li Liu, Hang Sun, Yunqiu Luo

**Affiliations:** ^1^Department of Endocrinology, The Second Affiliated Hospital of Chongqing Medical University, Chongqing, China; ^2^Second Clinical College, Chongqing Medical University, Chongqing, China

**Keywords:** diabetic foot, nutritional screening, Mini-Nutritional Assessment short form, Nutritional Risk Screening 2002, GLIM, malnutrition

## Abstract

**Background:**

Malnutrition has numerous adverse effects on the treatment and prognosis of diabetic foot (DF) patients, making it essential to determine the nutritional state to recognize malnutrition as early as possible. However, there is currently no acknowledged nutritional screening instrument for DF patients. This research aimed to identify the most appropriate nutritional assessment tool for this population.

**Methods:**

We conducted a cross-sectional study with 247 DF patients. Nutritional assessments were performed using Nutritional Risk Screening 2002 (NRS2002) and the Mini-Nutritional Assessment short form (MNA-SF). The comparisons between scales were carried out based on the Global Leadership Initiative on Malnutrition (GLIM) criteria. The Cohen's kappa (*k*) and the area under the receiver operating characteristic curve (AUC) were analyzed to measure the diagnostic agreement of malnutrition among the screening tools and the GLIM criteria.

**Results:**

Ninety-eight patients (39.68%) were diagnosed with malnutrition according to the GLIM criteria. The detection rates of MNA-SF and NRS2002 were 48.18 and 42.51%, respectively. MNA-SF was better correlated with the GLIM criteria, with a higher Kappa value (0.665 vs. 0.535) and a greater area under the receiver operating characteristic curve (0.860 vs. 0.792) than NRS2002. Additionally, MNA-SF and NRS2002 had similar specificity (79.2 vs. 85.2%), but MNA-SF demonstrated higher sensitivity (89.8 vs. 67.4%).

**Conclusions:**

This study is the first to describe the malnutrition diagnostic capacity of nutritional screening tools (MNA-SF and NRS2002) compared with the GLIM criteria. Our results indicate that the incidence of malnutrition is relatively high among DF patients, and the MNA-SF showed better sensitivity and correlation with the GLIM diagnostic criteria for malnutrition than NRS2002. Therefore, MNA-SF is more recommended for screening malnutrition in the DF population.

## Introduction

Diabetic foot (DF), which refers to the infection, ulceration, and (or) deep tissue destruction of the feet caused by varying degrees of nerve abnormalities and vascular lesions in the distal lower extremities, is one of the most severe complications and a common cause of admission (readmission) for patients with diabetes mellitus (DM) (1, 2). The global average prevalence of DF is reported to be 6.4%, while in China, it is 8.1% (3, 4), and about 25% of diabetic patients would develop DF during their lifetime (1). According to the guidelines of the European Society for Clinical Nutrition and Metabolism (ESPEN), malnutrition can be defined as the adverse effect of existing or potential nutritional and metabolic conditions on clinical outcomes related to diseases or surgeries (5), and it has been regarded as a major public global health problem. However, because the signs and symptoms are often subtle, malnutrition are frequently overlooked by clinical staff, despite their significant economic burden. Previous studies have shown that nutritional status is closely related to the presence and development of DF (6, 7). Moreover, malnutrition is more common among DF patients compared to non-DF patients (8). Once malnutrition occurs in DF patients, it brings a series of hazards, such as decreased immune function, increased infection rates, and a higher likelihood of amputation, all of which greatly delay wound healing, prolong hospitalization, and significantly reduce quality of life (9, 10).

Given the adverse impacts of malnutrition on the treatment, prognosis, and economic burden of DF patients, the implementation of early screening to correctly recognize is essential. Since the 1990s, scholars have developed numerous nutritional (risk) screening tools, and the standardized application of these tools has received increasing attention in recent years. The 2002 guidelines of the ESPEN recommend that different nutritional screening tools should be applied based on specific characteristics (11). For example, Nutritional Risk Screening 2002 (NRS2002) is recommended for hospitalized patients (12), the Mini Nutritional Assessment (MNA) for elderly patients, and the Malnutrition Universal Screening Tool (MUST) for adults living in the community (13, 14). Additionally, Fontes et al. (15) have demonstrated that the subjective global assessment (SGA) is considered a reliable tool for predicting outcomes in critically ill patients. Although the aforementioned scales have been broadly used in many healthcare settings, no reference has comprehensively documented the malnutrition diagnostic capacity of nutritional tools for DF patients. Thus, a validated nutritional screening instrument for this population is still a topic of discussion.

In 2019, the Global Leadership Initiative on Malnutrition (GLIM) guidelines were published and widely applied in clinical practice (16), these guidelines were established to reach a global consensus on the clinical diagnosis of malnutrition and aimed to promote international comparisons of the prevalence of malnutrition and the effectiveness of nutritional interventions. What's more, GLIM supports its position as the global core standard for the diagnosis of adult malnutrition with its uniformity, standardization, flexibility, and extensive validation and application. To our knowledge, Yuan et al. (17) have compared the prevalence of malnutrition in patients with DF between the GLIM criteria and SGA. Thus, the purpose of the current study was to describe the effectiveness of the diagnostic performances of two frequently used nutritional screening tools domestically (NRS2002 and Mini Nutritional Assessment short form) among DF patients, using the new GLIM criteria as the gold standard for malnutrition.

## Methods

### Study design and participants

This cross-sectional study was conducted between May 2021 and June 2024, with participants recruited from a hospital in Chongqing, China. Eligibility criteria included a diagnosis of diabetic DF, age 18 years or older, stage 1–5 of the DF ulcer measured by the Wagner–Merrit classification system (18), and receiving medications for glycemic control. Patients were excluded if they were pregnant, had serious acute complications such as diabetic ketoacidosis, concomitant tumors at any phase, tuberculosis, hyperthyroidism, or a history of mental illness that prevented them from completing the nutritional assessment. With an effect size of 0.5 and a Type I error rate of 0.05, power = 0.80, and a sampling ratio of 1:1, the minimum patient sample size calculated by the G^*^Power 3.1 software was 164. This study was approved by the Ethics Committee of the Second Affiliated Hospital of Chongqing Medical University [NO: 2022 Coronation Review No. (69)]. Each participant was informed of the study's purpose, and verbal or written informed consent was obtained. The study protocol adhered to principles of anonymity and confidentiality.

### Data collection

Participants' demographic factors, including gender, age, smoking and alcohol history, type of nutritional consumed, and education level were self-reported at the baseline assessment. Clinical and laboratory variables were gathered through the electronic medical record system, including body mass index (BMI), comorbidities, duration of diabetes and DF, diabetes treatment modality, Wagner grade of DF, presence of infection in the foot ulcer, serum C-reactive protein (CRP), triglyceride, hemoglobin A1c, hemoglobin, albumin, and glomerular filtration rate. BMI was calculated as weight (kg) divided by height (m)^2^. Anthropometric measurements were collected at 7 a.m., on the day after patients' admissions with an empty stomach, and patients were requested to wear hospital uniforms without shoes for evaluation. Body weight and height were measured on a scale (SECA 799) to the nearest 0.1 kg and 0.1 cm three consecutive times, and the mean values were adopted. For participants unable to stand, knee length was used to estimate height and wheelchair scales were used to measure weight (17). Data collectors were all diabetes specialist nurses who were not informed of the study's goal in advance to minimize bias in the data collection process. Laboratory measurements were examined in blood samples gathered the morning after admission.

### Nutritional assessment

The NRS2002 consists of three sections: severity of disease, impaired nutritional status, and age (12). The highest score is seven, with the first two sections ranging from 0 to 3, and an additional point added if the patient is over 70 years old. The scores are classified as 0–2 points indicating normal status and 3–7 points indicating malnutrition. The percentage of unintentional weight loss over the last 3 months were calculated from patients' or their caregivers' reports.

The Mini Nutritional Assessment short form (MNA-SF) is a simplified version of the MNA (19), characterized by higher sensitivity, specificity, and shorter time consumption compared to the MNA. The MNA-SF comprises six domains: BMI, appetite or other eating problems in the past 3 months, weight loss, movement impairment, acute illness or stress, and dementia or depression. The overall score of the MNA-SF is 14 points, 0–11 points indicating malnourishment and 12–14 points indicating normal nutritional status.

A two-step approach for the GLIM criteria for the malnutrition diagnosis was performed, firstly screening status by the use of NRS2002, and further, evaluating patients with NRS2002 ≥3 points, the diagnosis of malnutrition should meet at least one phenotypic criterion and one etiologic criterion (16). The phenotypic criteria include weight loss, low BMI, and reduced muscle mass, while the etiologic criteria include reduced food intake or assimilation and chronic inflammation. Unintentional weight loss is considered significant if there is a weight loss of more than 5% within the past 6 months or more than 10% beyond 6 months. Low BMI is identified as BMI < 18.5 kg/m^2^ for those under 70 years old and BMI < 20.0 kg/m^2^ for those over 70 years old. For reduced muscle mass, since there is no unified standard in China, the Japanese standard for sarcopenia was referenced in this study. Muscle loss is defined as calf circumference (CC) ≤ 30 cm for males and ≤ 29 cm for females (20). As DF meets the etiologic criteria for disease-related inflammation, CRP > 10 mg/L was considered a supportive indicator of inflammation (21). All participants completed nutritional status assessments by a proficient nutritionist in the same order within 48 h of their admissions. And in order to prevent measurement bias, we blinded the evaluators in comparing the tools and applying the GLIM standards.

### Statistical analysis

Statistical analysis was performed using SPSS Statistics software version 26.0 (Armonk, NY, USA). Normally distributed continuous variables were presented as mean ± standard deviations (SD), and categorical variables were expressed as frequencies and percentages. The Student's *t*-test and chi-square test were used to explore differences in baseline characteristics between the two groups. Cohen's kappa (*k*) coefficient was used to measure the diagnostic agreement of malnutrition among the screening tools and the GLIM criteria. A kappa value of 0, 0.01–0.20, 0.21–0.40, 0.41–0.60, 0.61–0.80, and 0.81–1.00 represents no, slight, fair, moderate, substantial, and almost perfect agreement, respectively (22). The area under the receiver operating characteristic curve (AUC) was used to evaluate the ability of the tools to distinguish between malnourished and non-malnourished patients. AUC ranges from 0 to 1, with accuracy considered high when AUC is >0.90, moderate from 0.70 to 0.90, and low from 0.50 to 0.69. Additionally, sensitivity, specificity, positive and negative predictive values, and positive (LR+) and negative (LR–) likelihood ratios were calculated to describe the performance of the tools. All reported *p*-values are based on two-sided tests, with a significance level of 5%.

## Results

### Participants characteristics

Among the 262 eligible participants, 15 patients were excluded due to tuberculosis (two patients), being on dialysis (three patients), a history of mental illness (three patients), and unwillingness to participate (seven patients). Thus, 247 participants were enrolled in the study and completed the assessment. The cohort consisted of 150 men and 97 women, with a mean age of 66.77 ± 13.61 years. The characteristics of the sample are presented in Table 1. There were no significant differences in the rates of malnutrition among DF patients with different ages, smoking and alcohol history, education levels, comorbidities, diabetes treatment, CRP, triglyceride and glomerular filtration rates.

**Table 1:** The characteristics of the patients according to the GLIM criteria (*n* = 247).

**Items**	**Malnourished (*n* = 98)**	**Normal (*n* = 149)**	***p***
	**Mean** ±**SD or** ***n*** **(%)**	**Mean** ±**SD or** ***n*** **(%)**	
Age (years)	66.47 ± 14.06	66.96 ± 13.35	0.782
**Gender**			0.046
Male	67 (44.67)	83 (55.33)	
Female	31 (31.96)	66 (68.04)	
**Smoke**			0.238
No	36 (35.29)	66 (64.71)	
Yes	62 (42.76)	83 (57.24)	
**Alcohol**			0.190
No	41 (35.34)	75 (64.66)	
Yes	57 (43.51)	74 (56.49)	
**Comorbidities**			0.364
< 3	42 (36.52)	73 (63.48)	
≥3	56 (42.42)	76 (57.58)	
**Education level**			0.346
Primary school	41 (37.96)	67 (62.04)	
Middle school	34 (47.22)	38 (52.78)	
High school	15 (38.46)	24 (61.54)	
College	8 (28.57)	20 (71.43)	
**Type of nutritional intake**			0.032
General diet	55 (56.12)	81 (54.36)	
Soft diet	18 (18.37)	46 (30.87)	
Semi-liquid diet	18 (18.37)	12 (8.06)	
Liquid diet	7 (7.14)	10 (6.71)	
DM course (years)	13.85 ± 9.52	10.13 ± 8.40	0.001
**Duration of DF**			< 0.001
< 3 months	28 (25.23)	83 (74.77)	
≥3 months	70 (51.47)	66 (48.53)	
**CRP** ≥**10 mg/L**			0.436
No	19 (34.55)	36 (65.45)	
Yes	79 (41.15)	113 (58.85)	
BMI (kg/m^2^)	21.47 ± 2.70	23.28 ± 3.54	< 0.001
**Wagner grade**			< 0.001
1–2	14 (15.56)	76 (84.44)	
3–5	84 (53.50)	73 (46.50)	
**DF with infection**			< 0.001
No	35 (28.23)	89 (71.77)	
Yes	63 (51.22)	60 (48.78)	
**Diabetes treatment**0.0770.077		
Oral only	10 (26.32)	28 (73.68)	
Injection only	25 (53.19)	22 (46.81)	
Insulin pump	33 (41.25)	47 (58.75)	
Combined	30 (36.59)	52 (63.41)	
HbA1c (%)	9.71 ± 2.03	8.93 ± 1.87	0.002
CC (cm)	29.69 ± 7.16	31.60 ± 6.78	0.036
Triglyceride (mmol/L)	1.60 ± 1.12	1.63 ± 0.94	0.810
Hemoglobin (g/L)	121.21 ± 14.76	126.70 ± 11.58	0.001
Albumin (g/L)	37.90 ± 5.47	41.38 ± 3.71	< 0.001
GFR (ml/min)	93.09 ± 22.58	98.37 ± 23.15	0.078

SD, standard deviation; DM, diabetes mellitus; DF, diabetic foot; CRP, C reactive protein; BMI, body mass index; HbA1c, hemoglobin A1c; CC, calf circumference; GFR, glomerular filtration rate.

### Nutritional assessment

We found 142 people with normal nutrition and 105 with malnutrition by NRS2002. When employing the MNA-SF, we discovered 128 people with normal nutrition and 119 with malnutrition. According to the GLIM criteria, 98 participants were diagnosed malnutrition. The cross-tabulation of the results of nutritional screening with NRS2002 and MNA-SF and the classification of malnutrition according to the GLIM diagnostic criteria can be found in Table 2.

**Table 2:** Cross tabulation of the results of NRS2002, MNA-SF, and GLIM criteria for the diagnosis of malnutrition.

**Category**		**NRS2002 (** ***n*** **)**		**MNA-SF (** ***n*** **)**		**Total**
		**Normal**	**Malnourished**	**Normal**	**Malnourished**	
GLIM criteria (*n*)	Normal	118	31	118	31	149
	Malnourished	24	74	10	88	98
Total		142	105	128	119	247

### Statistical evaluation of nutritional screening tools compared to the GLIM criteria

We observed variations in the agreement between the GLIM diagnostic criteria for malnutrition and the two nutritional screening tools. Specifically, NRS2002 showed a lower agreement (*K* = 0.535, *p* < 0.001) with the GLIM criteria compared to MNA-SF (*K* = 0.665, *p* < 0.001). This indicates that the NRS2002 and MNA-SF scales have moderate and high abilities to screen for malnutrition in DF patients, respectively. Both scales demonstrated high specificity in identifying malnutrition according to the GLIM criteria, but the sensitivity of MNA-SF was slightly better than that of NRS2002 (89.8 vs. 67.4%). Additionally, MNA-SF had a higher negative predictive value, LR+, and a lower LR– compared to NRS2002. Furthermore, the superior ability of MNA-SF to distinguish malnourished patients was evidenced by its higher AUC value compared to NRS2002 (0.860 vs. 0.792). Detailed results are presented in Table 3.

**Table 3:** Statistical evaluation of the nutritional screening tools (MNA-SF and NRS2002) compared to the GLIM diagnostic criteria of malnutrition.

**Items**	**NRS2002**	**MNA-SF**
Sensitivity	67.4%	89.8%
Specificity	85.2%	79.2%
Positive predictive value	75.0%	74.0%
Negative predictive value	79.9%	92.2%
Positive likelihood ratio (LR+)	4.32	4.56
Negative likelihood ratio (LR–)	0.38	0.13
K value (*p*-value)	0.535 (< 0.001)	0.665 (< 0.001)
AUC (95% confidence interval)	0.792 (0.736, 0.841)	0.860 (0.810, 0.901)

## Discussion

Our study showed that 98 participants (39.68%) were diagnosed with malnutrition according to the GLIM criteria, aligning with other studies reporting prevalence rates of 24%−62% (8, 23, 24). The wide range in the assessed prevalence of malnutrition can be predominantly attributed to the lack of a unified definition of malnutrition, differences in nutritional screening instruments, and the characteristics of the individuals included. However, all studies consistently found a remarkably high prevalence of malnutrition among DF patients. It is clear that the current study provides valid and valuable evidence to emphasize the importance of early recognition of potential malnutrition. Therefore, we recommend that healthcare teams place more emphasis on assessing nutritional status rather than solely focusing on basic treatment planning, such as drug therapy and wound repair in DF patients.

The malnutrition rate we observed is lower than the 62% reported in Zhang et al.'s (8) study, which used SGA for diagnosis. A recent systematic review indicated that the SGA covers all aspects of the conceptual definitions of malnutrition (25), which may explain the higher proportion. Although SGA can identify existing risk factors for malnutrition, it is not recognized as a quick or convenient instrument for clinical practice and is considered complicated and time-consuming, typically taking nearly 10 min (9 ± 1.1 min) to complete assessments (26). Additionally, our study found that a higher proportion of patients were identified as malnourished using the MNA-SF compared to NRS2002, which is also observed in elderly populations (26). This evidence can be largely explained by the fact that MNA-SF considers more comprehensive conditions, such as psychological stress or acute disease within the past 3 months, impaired mobility and neuropsychological problems which NRS2002 does not include may indirectly affect nutritional status.

Our study supports that the incidence of malnutrition varies among patients with different durations of diabetes and DF, Wagner grade, BMI, and ulcer infection, which was similar to previous studies (24, 27). Moreover, age has been shown to be significantly correlated with nutritional status in prior research (28), older patients are more likely to suffer from malnutrition. The reason could be attributed to the truth that diabetic patients are prone to appear insulin deficiencies or resistance, as for elderly patients particularly those with multiple comorbidities whose insulin level has decreased significantly will inevitably lead to slower protein synthesis and reduced nutrient production, which is not conducive to the disease recovery, tissue regeneration, and ulcer healing. We were surprised to find that the incidence of malnutrition varies between genders, with men being more likely to experience malnutrition. This may be due to that approximately two-thirds of the men in our study smoked, and the adverse effects of nicotine on tissue perfusion and wound healing in diabetic foot ulcers have been confirmed (29).

Our results revealed that MNA-SF has a greater *K* value and LR+ compared to NRS2002, indicating that MNA-SF has better agreement and performance with the GLIM diagnostic criteria than NRS2002. Additionally, the higher AUC value for MNA-SF suggests that individuals at high risk of malnutrition are more likely to be correctly diagnosed as malnourished using MNA-SF. Another study conducted by Andersen et al. (30) has drawn that the MNA-SF is also suitable for the dynamic nutritional assessment following multifactorial interventions in patients. While the MNA-SF and NRS2002 had similar specificity, MNA-SF demonstrated higher sensitivity. Although there is no universally accepted standard to theoretically discriminate sensitivity and specificity, we consistently agree that over-diagnosing malnutrition is preferable to missing potential cases (26). Therefore, based on the principle of selecting malnutrition screening tools with high sensitivity, MNA-SF is more appropriate for nutrition screening in DF inpatients. Furthermore, a published study illustrated that MNA-SF scores were inversely correlated with both major and minor lower-extremity amputation rates (8), suggesting that MNA-SF is a strong independent predictor of outcomes in DF patients. The time required to apply each tool was not compared in our study, however, a former study revealed that the average time of MNA-SF and NRS2002 spent with one subject was 2 and 3 min, respectively (26). In summary, these diagnostic, predictive capabilities and quickest application may make MNA-SF an excellent candidate for malnutrition screening among DF subjects.

In this study, we selected GLIM as the criteria to diagnose malnutrition instead of the scales commonly used by other authors, which often lack sufficient objective indicators (8, 26). For example, the quantitative descriptions of unintentional reductions in appetite and food intake are often limited and include weight loss, speculated from patients' or caregivers' reports, which could introduce recall bias. Furthermore, studies have suggested that BMI may no longer be a reliable parameter for screening malnutrition as it does not always accurately reflect nutritional status, especially when patients experience fluid overload and edema (31, 32). Recently, a newly proposed international consensus document recommended that assessing reduced muscle mass may overcome the limitations associated with BMI and promote exploring the prevalence of malnutrition among specific populations in the future (16).

Some limitations of this study should be acknowledged. First, it was a single-center study, lacking external cohort or validation set to confirm the diagnostic performance of the tools, consequently, the generalization of results to other settings such as primary care or different geographic regions should be approached cautiously. Large-scale, high-quality longitudinal studies should be designed in the future. Besides, the use of an indirect measure of muscle mass (calf circumference), based on Japanese criteria and not validated for the Chinese population, may compromise the accuracy of GLIM's phenotypic classification. Additionally, while nutritional screening should ideally be dynamic and real-time, we only focused on baseline nutritional status without tracking dynamic indicators. Lastly, we only diagnosed malnutrition without grading severity and examining whether nutritional support or specialized treatment impacts screening results and nutritional status. Given the high prevalence of malnutrition among DF patients, intervention studies should be initiated to provide significant new information for the treatment and care of malnourished patients.

## Conclusions

According to our study, malnutrition is highly prevalent among DF patients, highlighting the imperative need for early nutritional assessment and personalized nutritional supplementation. Considering the high incidence of malnutrition in this population, it is crucial to identify a simple and effective nutritional screening instrument. Our findings suggested that the MNA-SF had better agreement with the GLIM diagnostic criteria for malnutrition and showed higher sensitivity and AUC compared to NRS2002. Therefore, MNA-SF is more strongly recommended than NRS2002 among this population.

## Data Availability

The raw data supporting the conclusions of this article will be made available by the authors, without undue reservation.
